# AabHLH112, a bHLH transcription factor, positively regulates sesquiterpenes biosynthesis in *Artemisia annua*

**DOI:** 10.3389/fpls.2022.973591

**Published:** 2022-09-02

**Authors:** Lien Xiang, Ping He, Guoping Shu, Mingyuan Yuan, Mengling Wen, Xiaozhong Lan, Zhihua Liao, Yueli Tang

**Affiliations:** ^1^College of Environmental Science and Engineering, China West Normal University, Nanchong, China; ^2^Integrative Science Center of Germplasm Creation in Western China (CHONGQING) Science City and Southwest University, Tibet Agriculture and Animal Husbandry College and Southwest University (TAAHC-SWU) Medicinal Plant Joint R&D Centre, School of Life Sciences, Southwest University, Chongqing, China; ^3^Chongqing Academy of Science and Technology, Chongqing, China; ^4^The Provincial and Ministerial Co-founded Collaborative Innovation Center for R&D in Tibet Characteristic Agricultural and Animal Husbandry Resources, Food Science College, Tibet Agriculture and Animal Husbandry University, Nyingchi, China

**Keywords:** *Artemisia annua*, AabHLH112, β-caryophyllene, *epi*-cedrol, β-farnesene, metabolic regulation

## Abstract

The bHLH transcription factors play important roles in the regulation of plant growth, development, and secondary metabolism. β-Caryophyllene, *epi*-cedrol, and β-farnesene, three kinds of sesquiterpenes mainly found in plants, are widely used as spice in the food industry and biological pesticides in agricultural production. Furthermore, they also have a significant value in the pharmaceutical industry. However, there is currently a lack of knowledge on the function of bHLH family TFs in β-caryophyllene, *epi*-cedrol, and β-farnesene biosynthesis. Here, we found that AabHLH112 transcription factor had a novel function to positively regulate β-carophyllene, *epi*-cedrol, and β-farnesene biosynthesis in *Artemisia annua*. Exogenous MeJA enhanced the expression of *AabHLH112* and genes of β-caryophyllene synthase (*CPS*), *epi-*cedrol synthase (*ECS*), and β-farnesene synthase (*BFS*), as well as sesquiterpenes content. Dual-LUC assay showed the activation of *AaCPS, AaECS*, and *AaBFS* promoters were enhanced by AabHLH112. Yeast one-hybrid assay showed AabHLH112 could bind to the G-box (CANNTG) *cis*-element in promoters of both *AaCPS* and *AaECS.* In addition, overexpression of *AabHLH112* in *A. annua* significantly elevated the expression levels of *AaCPS*, *AaECS*, and *AaBFS* as well as the contents of β-caryophyllene, *epi*-cedrol, and β-farnesene, while suppressing *AabHLH112* expression by RNAi reduced the expression of the three genes and the contents of the three sesquiterpenes. These results suggested that *AabHLH112* is a positive regulator of β-caryophyllene, *epi*-cedrol, and β-farnesene biosynthesis in *A. annua*.

## Introduction

*Artemisia annua*, a traditional Chinese medicinal plant, not only produces the well-known artemisinin, but also produces a number of sesquiterpenes that play an important role in plants’ stress resistance, communication, and growth regulation (Chadwick et al., 2013). β-caryophyllene, *epi*-cedrol, and *β-*farnesene, three kinds of sesquiterpenes, are used as spice in the food industry and biological pesticides in agricultural production (Yu X. et al., 2012; Cheng et al., 2022). Besides, they also have a significant value in the pharmaceutical industry (Cheng et al., 2022). β-Caryophyllene was found to has good curative effect on colitis (Bento et al., 2011), cerebral ischemia (Chang et al., 2013), diabetes (Basha and Sankaranarayanan, 2014), anxiety and depression (Bahi et al., 2014), liver fibrosis (Mahmoud et al., 2014), and osteoarthritis (Rufino et al., 2015). In cancer studies, β-caryophyllene demonstrated synergy with the chemotherapy drug paclitaxel on human tumor cell lines, and alone it stimulates apoptosis and suppresses tumor growth (Legault and Pichette, 2007). *epi*-Cedrol is widely used in pharmaceutical industry for its sedative, anti-inflammatory, and cytotoxic activities (Zhang et al., 2016; Luo et al., 2019). β-farnesene, an important acyclic and volatile sesquiterpenes acting as the main component of the aphid alarm pheromones for many pest aphids, can be used to create new biological pesticides (Wang et al., 2014). Because of their pharmacological activity and commercial value, numerous studies have focused on improving the production of sesquiterpenes in *A. annua*.

**FIGURE 1:**
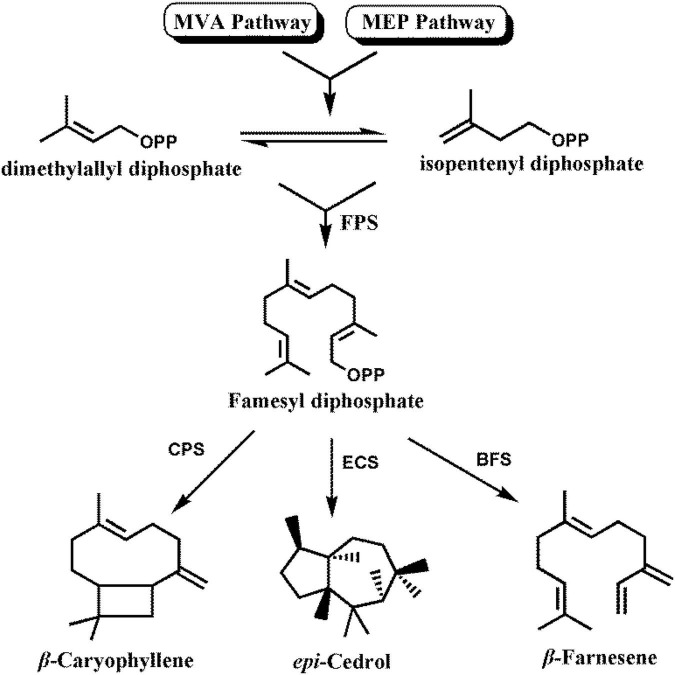
Biosynthetic pathway for sesquiterpenes in *Artemisia annua*. FPS, farnesyl diphosphate synthase; CPS, β-caryophyllene synthase; ECS, *epi*-cedrol synthase; BFS, β-farnesene synthase.

β-Caryophyllene, *epi*-cedrol, and β-farnesene are biosynthesized *via* both the cytoplasmic mevalonate (MVA) pathway and plastidial methylerythritol phosphate (MEP) pathway (Navale et al., 2019). Plant cells use one molecule of dimethylallyl diphosphate (DMAPP) and two molecules of isopentenyl diphosphate (IPP) to produce farnesyl diphosphate (FPP) in a reaction catalyzed by FPP synthase (FPS) (Navale et al., 2019). Then the sesquiterpene synthase takes FPP as the substrate to synthesize a variety of sesquiterpenes (Figure 1).

Transcription factors (TFs) are sequence-specific DNA-binding proteins which interact with the promoter regions of target genes and regulate their transcription (Yang et al., 2012). It is a useful tool to improve the production of secondary metabolites in pharmaceutically important plants, such as flavonoids or alkaloids (Gantet and Memelink, 2002). Recently, a number of AP2/ERF, WRKY, and bHLH transcription factors have been found to have global regulatory functions in pharmaceutical terpenoids biosynthesis (Lu et al., 2016; Li et al., 2017). In *A. annua*, overexpression of *AaERF1* and *AaERF2* significantly promoted the transcription of *ADS* and *CYP71AV1*, resulting in an increase of artemisinin content (Yu Z. et al., 2012). Overexpression of *SmERF1L1* significantly increased tanshinones production in transgenic *Salvia miltiorrhiza* hairy roots (Huang et al., 2019). In addition, overexpression of *SmWRKY1* significantly elevated the transcription of *SmDXS* and *SmDXR*, increasing tanshinone production in *S. miltiorrhiza* (Cao et al., 2018).

The bHLH family is the second largest transcription factor family in plants, which plays an important role in the regulation of pharmaceutical terpenoids biosynthesis (Ji et al., 2014). It was reported that *SmbHLH3* acts as a transcription repressor for both phenolic acids and tanshinones biosynthesis in *S. miltiorrhiza* hairy roots (Zhang et al., 2020). In *Arabidopsis*, AtMYC2, a JA-responsive TF, can enhance sesquiterpenes biosynthesis by binding to the promoters of *TPS11* and *TPS21* (Hong et al., 2012). Chuang et al. (2018) reported that overexpression of *PbbHLH4* markedly increased terpenes content in *Phalaenopsis*. JA-responsive *SlMYC1* differentially regulates monoterpene and sesquiterpenes biosynthesis in tomato, which positively regulates monoterpene biosynthesis in leaf and stem but negatively regulates sesquiterpenes biosynthesis in stem (Xu et al., 2018). *CrMYC2* directly activates the expression of *ORCA* genes through binding to the G-box in its promoter, and promotes vinblastine and vincristine biosynthesis in *Catharanthus roseus* (Zhang et al., 2011). *BpMYC4* and *BpbHLH9* from *Betula platyphylla Suk* are involved in regulating the biosynthesis of triterpenes (Yin et al., 2017). Furthermore, studies also showed that bHLH TFs could act as negative regulators in terpenes biosynthesis, including the biosynthesis of the diterpene paclitaxel and monoterpenoid indole alkaloid in *Taxus cuspidata* (Lenka et al., 2015) and *Catharanthus roseus* (Patra et al., 2018), respectively. One hundred and twenty two putative bHLH TFs were found in *A. annua* by genome-wide identification (Xiang et al., 2019) and only three bHLH TFs-*AabHLH1*, *AabHLH112*, and *AaMYC2*, respectively, have been reported to play regulatory roles in artemisinin biosynthesis (Ji et al., 2014; Shen et al., 2016; Xiang et al., 2019). Now, although our knowledge of the regulation of artemisinin biosynthesis is increasing, little is known about the regulation of non-artemisinin sesquiterpenes biosynthesis in *A. annua*. In this study, we found that the *AabHLH112* has a novel function in improving the accumulation of β-caryophyllene, *epi*-cedrol, and β-farnesene in *A. annua*, and revealed the mechanism of AabHLH112-mediated upregulation of sesquiterpenes biosynthesis. Our results suggested that this transcription factor could be a useful tool to improve sesquiterpenes production in *A. annua*.

## Materials and methods

### Plant material and growth conditions

Seeds were harvested from wild-type *A. annua* grown in the experimental field of Southwest University (Chongqing, China) for this study. These seeds were surface-sterilized with 20% sodium hypochlorite solution for 20 min, and then washed three times with sterile water. Subsequently, seeds were germinated on 1/2 MS solid medium at 23 ± 2°C under a light period of 16-h-light/8-h-dark. All seedlings were grown in pots with organic substrates in an artificial climate room at 23 ± 2°C under a light period of 16-h-light/8-h-dark. *Nicotiana benthamiana* seeds were sown directly on solid and their growth conditions are consistent with *A. annua*. *Nicotiana benthamiana* plants grown for 30 days were used for dual-luciferase assays.

### Exogenous methyl jasmonate treatment and sample collection

One-month old wild-type *A. annua* plants were treated according to the report (Xiang et al., 2015) with modifications. 300 μM exogenous MeJA solutions containing 0.4% ethanol was sprayed on the plant surfaces. Then the leaves of *A. annua* plants at 0, 3, 6, 12, and 24 h after exogenous MeJA treatment were collected respectively and then put in liquid nitrogen immediately for RNA extraction and determination of sesquiterpenes contents.

### *Artemisia annua* transformation

The coding sequence of *AabHLH112* was amplified and inserted into the pHB binary plasmid vector through *Bam*HI and *Pst*I sites to construct the plant overexpression vector, pHB-AabHLH112. Besides, the 352bp fragment from the *AabHLH112* was amplified and inserted into the intermediate pHANNIBAL vector, and then the expression cassette was recombined into the pBin19 plasmid for constructing the *AabHLH112*-RNAi vector. pHB-AabHLH112 and pBin19-RNAi-AabHLH112 vectors were transferred into *A. tumefaciens* strain EHA105 to form engineering strains. These strains were grown on YEP solid medium containing related antibiotics for 48 h. Subsequently, positive monoclonal strain was inoculated into YEP liquid medium containing related antibiotics to culture OD_600_ = 0.5∼0.6. The supernatant was discarded after centrifugation, and then resuspended in the 1/2 MS liquid to OD_600_ = 0.3∼0.5. Then the culture was shaken at 200 rpm/28°C for 30 min. Cultured engineered strains were used to transform *A. annua via Agrobacterium*-mediated transformation as described previously (Xiang et al., 2019). After that, the transformed seedlings were transplanted into pots with organic substrates and cultured in an artificial climate room at 23 ± 2°C under a light period of 16 h-light/8 h-dark.

### Quantitative real-time polymerase chain reaction

Quantitative real-time PCR was performed to analyze genes’ expression in this study. Total RNA of root, stem, leaf and flower was extracted using the total plant RNA Extract Kit (Tiangen, China), and then reversely transcribed into cDNA using Fast King RT Kit (Tiangen, China). Subsequently, the cDNA was used as the template for detecting the expression levels of *AabHLH112* and sesquiterpene synthase genes *AaCPS*, *AaECS*, and *AaBFS* by qPCR experiment. The qPCR amplification conditions were 95°C for 3 min, followed by 40 cycles of 95°C for 15 s, 55°C for 20 s, and 72°C for 20 s. *β-actin* of *A. annua* was used as the reference gene in this study (Ma et al., 2018), and the relative expression levels were calculated using the 2^–△△Ct^ method (Livak and Schmittgen, 2001). In addition, the expression levels of sesquiterpenes-related genes were detected in *AabHLH112*-overexpressing and RNAi-*AabHLH112* transgenic *A. annua* using the above method. The primer sequences in qPCR experiment were listed in Supplementary Table 1.

### Plant sesquiterpenes extraction and gas chromatography-mass spectrometry analysis

The sesquiterpenes of *A. annua* leaves were analyzed by gas chromatography-mass spectrometry (GC-MS) as described before with some modifications (Fu et al., 2017). The fresh leaves 1–8 from the main stem of 3 months old *AabHLH112*-overexpressing transgenic and wild-type *A. annua* plants were harvested, frozen immediately in liquid nitrogen, and then freeze-dried for 72 h at -80°C. 100 mg leaf powder was soaked in 4 mL *n*-hexane in 15 mL centrifuge tube with 35 μL trans-farnesol (80 μg/mL) as the internal standard, and ultrasonically extracted for 45 min at 28°C in an ultrasonic processor (KQ-500DE; Kunshan Ultrasonic Instrument Co. Ltd., Kunshan, China). Plant extract was centrifuged at 1,000 rpm for 10 min. The supernatant was filtered through 0.22 μm pore size filters and then analyzed by gas chromatography-mass spectrometry (GCMS-QP2010 Ultra; Shimadzu) with the temperature program: initial temperature of 70°C (1 min hold), increase to 160°C at 10°C/min, ramp to 240°C at 5°C/min, and finally increase to 280°C at 20°C/min (17 min hold). Helium was used as a carrier gas and 1 μL sample was injected in split mode; split rate, 2:1; ion source temperature, 230°C; ionization voltage, 70 eV with scanning from *m/z* 33 to 500. Qualitative analysis of all compounds was fulfilled by comparing with NIST (National Institute of Standards and Technology) database and Wiley libraries. The relative contents of sesquiterpenes were calculated by comparing peak areas with that of the internal standards. Trans-farnesol was purchased from Sigma-Aldrich in this study.

### Cloning and analysis of the promoter regions of sesquiterpene synthase genes

To isolate the promoter fragments of sesquiterpene synthase genes (*AaCPS*, *AaECS*, and *AaBFS*), the total DNA of *A. annua* leaves was extracted as the template using cetyltrimethylammonium bromide (CTAB) method. All primer sequences were designed according to the genome of *A. annua*. To avoid non-specific amplification, forward and reverse primer sequences are located in their promoter and ORF regions, respectively. These promoter fragments were obtained by nested PCR using the Pro Taq DNA polymerase (Aikerui, Changsha, China) according to the manufacturer’s instructions. Agarose gel electrophoresis was used to detect PCR products. Subsequently, these fragments were inserted into the pJET2.1 vector (Thermo Fisher Scientific, Waltham, MA, United States) and sequenced respectively. The *cis*-elements of promoter region in *AaCPS*, *AaECS*, and *AaBFS* were predicted using plantCARE online website.^1^ The promoter sequences of sesquiterpene synthase genes were shown in Supplementary Data Sheet 1. Primers were listed in Supplementary Table 1.

### Dual-luciferase assay

Dual-LUC assays were performed using the methods reported previously (Xiang et al., 2019). The promoter sequences of *AaCPS* (OP056317), *AaECS* (OP056318), and *AaBFS* (OP056319) genes were inserted into pGreenII 0800-LUC plasmid to generate pAaCPS:LUC, pAaECS:LUC, and pAaBFS:LUC constructs as the reporter vector, respectively. Subsequently, these reporter vectors were transferred into *A. tumefaciens* strain GV3101 together with the pSoup plasmid. The *AabHLH112* (MG872820) was inserted into the pHB plasmid driven by CaMV 35S promoter as the effector vector and transferred into *A. tumefaciens* strain GV3101. Meanwhile, the pHB-YFP plasmid was transferred into GV3101 as a negative control. All engineering and control strains were inoculated into YEP liquid selective medium and cultured overnight at 28°C. The agrobacterium cells were collected by centrifuge at 4,500 rpm for 10 min and resuspended in the MS liquid to OD_600_ = 0.6 ± 0.05. The acetosyringone (As, 100 mM, 1:500, v:v) and 2-(*N*-morpholino) ethanesulfonic acid [MES, 0.5 M (pH = 5.7), 1:50, v:v] were added to the resuspension and then were injected into tobacco leaves after being placed for 4 h at room temperature. Tobacco plants injected with agrobacterium cells were exposed to weak light for 48–72 h. Infiltration and detection were performed as described previously (Ma et al., 2018). All experiments were repeated five times for each combination. Primers are listed in Supplementary Table 1.

### Yeast one-hybrid assay

To investigate how AabHLH112 regulates the expression of *AacCPS*, *AaECS*, and *AaBFS*, yeast one-hybrid assays were fulfilled as described previously (Xiang et al., 2019). The coding sequence of *AabHLH112* was amplified and inserted into pB42AD plasmid with the GAL4 activation domain (AD) through *Eco*RI and *Xho*I sites to generate pB42AD*-AabHLH112* constructs as the prey. The 45∼55 bp fragments containing one G-box motifs from *AaCPS*, *AaECS*, and *AaBFS* promoters, named pAaCPS-G1 (−884∼−840), pAaCPS-G2 (−838∼−794), pAaCPS-G3 (−806∼−762), pAaCPS-G4 (−430∼−386), pAaECS-G1 (−1228∼−1178), pAaECS-G2 (−915∼−866), pAaECS-G3 (−385∼−336), pAaBFS-G1 (−1045∼−996), pAaBFS-G2 (−985∼−935), pAaBFS-G3 (−66∼−15) and the mutant of G-box were inserted into pLacZ plasmid through *Kpn*I and *Xho*I sites as the bait, respectively. The pB42AD-*AabHLH112* plasmid was co-transformed into yeast strain EGY48 with each individually bait constructs, respectively. The yeast cells were grown on SD-Ura-Trp selective medium for 48 h at 30°C. All independent yeast cells were shifted into SD-Ura-Trp liquid medium and cultured overnight at 30°C, and then these cells were collected by microcentrifugation and resuspended in 100 μL sterile water. Resuspended cells were grown on SD/-Ura-Trp medium with 5-Bromo-4-chloro-3-indolyl-β-D-galactopyranoside (X-gal) for 24 48 h at 30°C. The empty pB42AD and pLacZ plasmids were used as the negative control. Five independent biological replicates were implemented for each experiment in this study. The primers are listed in Supplementary Table 1.

## Results

### Tissues expression patterns of AabHLH112 and three sesquiterpene synthase genes

**FIGURE 2:**
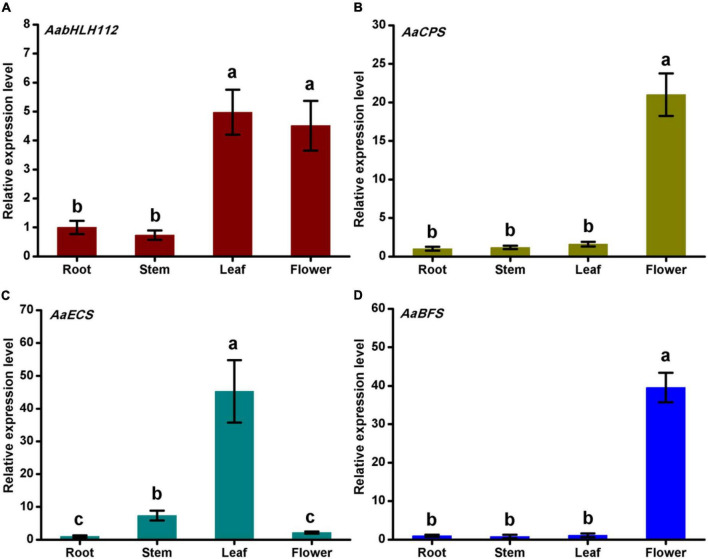
Genes expression levels of *AabHLH112* **(A)**, *AaCPS* **(B)**, AaECS **(C)**, and AaBFS **(D)** in root, stem, leaf, and flower of *Artemisia annua*. Bars are means ± SD from three independent biological replicates. One-way ANOVA was tested for significant differences among the means (indicated by different letters at *p* < 0.01).

To study the tissue expression patterns of *AabHLH112* and sesquiterpenes biosynthesis genes, we measured their expression in various *A. annua* tissues including roots, stems, leaves, and flowers. *AabHLH112* was highly expressed in flowers and leaves, and lowly expressed in roots and stems (Figure 2A). *AaCPS* and *AaBFS* genes’ expression has a tissue-specific mode, with a high expression in flowers and low expression in leaves, stems, and roots (Figures 2B,D). By comparison, *AaECS* was highly expressed in leaves and lowly expressed in roots and flowers (Figure 2C). Together, *AabHLH112*, *AaCPS*, *AaECS*, and *AaBFS* were expressed in all of the detected tissues.

### Methyl jasmonate treatment induced the expression of *AabHLH112* and three sesquiterpene synthase genes

**FIGURE 3:**
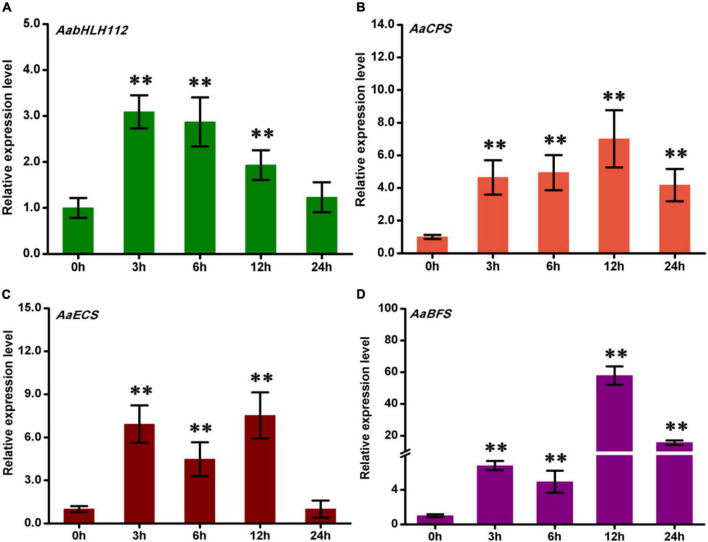
The expression of *AabHLH112* **(A)**, *AaCPS* **(B)**, *AaECS* **(C)**, and *AaBFS* **(D)** in response to MeJA treatment. Error bars represent the standard deviations of three technical replicates. Statistical significance was assessed with Student’s *t*-test (**p < 0.01).

The phytohormone JA plays a critical role in plant stress resistance and secondary metabolism (Wasternack and Song, 2017; Kianersi et al., 2021). To investigate the response of *AabHLH112*, *AaCPS*, *AaECS*, and *AaBFS* to MeJA treatment, wild type *A*. *annua* plants were treated with exogenous MeJA, and all the genes’ expression was quantified by qPCR. The expression of *AabHLH112* was induced by MeJA, rapidly increased and peaked after 3 h of treatment, followed by a gradual decrease (Figure 3A). This result indicated that *AabHLH112* is involved in MeJA response in *A*. *annua*, as in the case with the *Arabidopsis* homologous *AtbHLH33* (Li et al., 2020). Several studies have shown that the expression of many genes involved in terpenes biosynthesis is significantly increased under JA treatment. Here, we found the expression levels of *AaECS*, *AaCPS*, and *AaBFS* were significantly upregulated under exogenous MeJA treatment (Figures 3B–D); besides, the accumulation of β-caryophyllene, *epi*-cedrol, and β-farnesene, direct products of the three enzymes, also showed an upward trend after MeJA treatment (Supplementary Figure 1). These results suggest that MeJA can induce the expression of these sesquiterpenes synthase genes, thus enhancing the biosynthesis of β-caryophyllene, *epi*-cedrol and β-farnesene in *A*. *annua*.

### AabHLH112 activates the expression of the *AaECS*, *AaCPS*, and *AaBFS*

**FIGURE 4:**
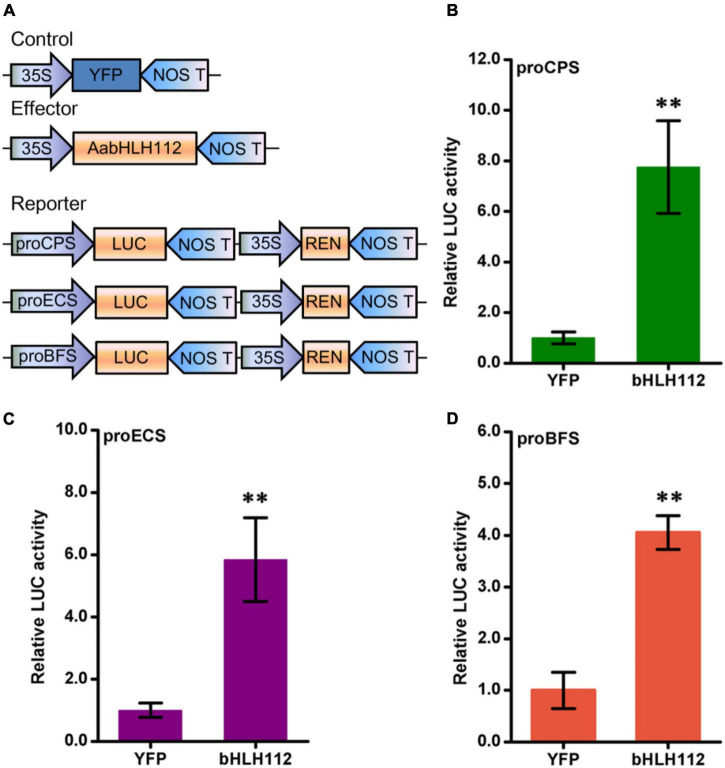
Biochemical assays between AabHLH112 and sesquiterpenes synthase genes. **(A)** Diagrams of reporter and effector constructs in transient dual-luciferase assays (REN, renilla luciferase; LUC, firefly luciferase). **(B–D)** Effects of AabHLH112 on activities of the *AaCPS*, *AaECS*, and *AaBFS* promoters in *Nicotiana benthamiana* leaves. The YFP effector was used as a negative control. The data represents the means ± SD (*n* = 3), ^**^p < 0.01 in student’s *t*-test.

Dual-LUC assays can be used to study the transcriptional regulation of transcription factors on target genes at the transcriptional level (Ma et al., 2018). The coding sequence of *AabHLH112* was inserted into the plant overexpression vector pHB as an effector construct, and the promoter sequences of *AaCPS* (Supplementary Figure 2), *AaECS* (Supplementary Figure 3), and *AaBFS* (Supplementary Figure 4) genes were inserted into pGreenII 0800-LUC plasmid to generate pAaCPS:LUC, pAaECS:LUC, and pAaBFS:LUC constructs as the reporter vector, respectively. Meanwhile, the pHB-YFP (yellow fluorescent protein) plasmid was used as a negative control (Figure 4A). The effector and reporter constructs were transiently expressed in *N. benthamiana* leaves using *Agrobacterium tumefaciens*-mediated co-infiltration. The dual-LUC assay results showed that the promoter activity of the above sesquiterpene synthase genes *AaCPS*, *AaECS*, and *AaBFS* was significantly increased, with the LUC/REN value increased by 7.75, 5.58, and 4.05-folds than the YFP control (Figures 4B–D), respectively.

### AabHLH112 bind to G-box *cis*-elements in the promoters of *AaCPS* and *AaECS*

**FIGURE 5:**
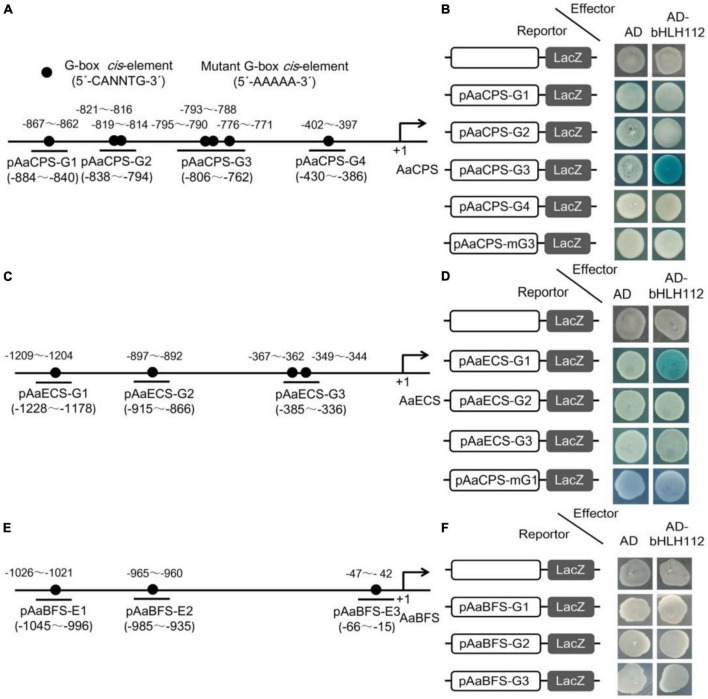
Yeast one-hybrid assays between AabHLH112 and G-box *cis*-elements of sesquiterpene synthase gene promoters, blue plaques indicate protein-DNA interactions. **(A)** Schematic diagram of the *AaCPS* promoter; **(B)** Y1H assay between AabHLH112 and *AaCPS* promoter; **(C)** Schematic diagram of the *AaECS* promoter; **(D)** Y1H assay between AabHLH112 and *AaECS* promoter; **(E)** Schematic diagram of the *AaBFS* promoter; **(F)** Y1H assay between AabHLH112 and *AaBFS* promoter; the black circles were the potential G-box elements in three promoters.

The core DNA sequence motif recognized by bHLH proteins is a consensus hexanucleotide sequence known as the G-box (5-CANNTG-3). It was previously reported that AaMYC2 can specifically bind to the G-box *cis*-elements in the promoters of key enzyme genes of artemisinin biosynthesis, including *AaCYP71AV1* and *AaDBR2* (Shen et al., 2016). Recently, Xiang et al. (2019) reported that AabHLH112 can directly bind to G-box in the *AaERF1* promoter and promote artemisinin biosynthesis. The above dual-LUC results showed that AabHLH112 could activate the transcription of *AaCPS*, *AaECS*, and *AaBFS*. In order to further investigate how AabHLH112 regulates the transcription of these sesquiterpene synthase genes, the promoter sequences of *AaCPS*, *AaECS*, and *AaBFS* were analysed by PlantCARE online website, and multiple G-box motifs were found in each promoter (Figures 5A,C,E). Following that, yeast one-hybrid assay was used to evaluate whether AabHLH112 can directly bind to the G-box motifs in these promoters. 45 50 bp fragments containing G-box motifs from each promoter were inserted into pLacZ plasmid as the bait, and *AabHLH112* was inserted into pB42AD plasmid with the GAL4 activation domain to generate pB42AD*-AabHLH112* constructs as the prey. The results indicated that AabHLH112 can directly bind to pAaCPS-G3 and pAaECS-G1 fragments containing the G-box in *AaCPS* and *AaECS* promoters (Figures 5B,D), but not the pAaCPS1-G1, pAaCPS-G2, pAaCPS-G4, pAaECS-G2, pAaECS-G3, and pAaBFS-G1, pAaBFS-G2, pAaBFS-G3 fragments. When the G-box *cis*-elements in the pAaCPS-G3 and pAaECS-G1 fragments were mutated to the 5’-AAAAA-3’ sequences resulting in pAaCPS-mG3 and pAaECS-mG1, respectively, the binding signals of AabHLH112 and pAaCPS-mG3 and pAaECS-mG1 disappeared (Figures 5B,D). These results demonstrate that AabHLH112 enhances *AaCPS* and *AaECS* transcription by directly binding to the specific G-box *cis*-elements in their promoters.

### Generation of *AabHLH112*-overexpression and RNAi trangenic *Artemisia annua*

To better explore the function of *AabHLH112* in sesquiterpenes biosynthesis in *A. annua*, *AabHLH112*-overexpressing and *AabHLH112*-RNAi transgenic *A. annua* plants were generated (Supplementary Figure 5). Primers designed according to the sequences of rubisco gene (*rbc*), *AabHLH112* and marker gene (hygromycin-resistant) were used to determine the regeneration plants by genomic PCR. The 877bp fragment of the hygromycin-resistant gene and coding sequence of AabHLH112 with partial *rbc* terminator (1383bp) were specifically amplified from the transformed plants and the positive control (pHB-*AabHLH112*), while these specific fragments could not be amplified from wild-type plants (Supplementary Figures 5B,C). Besides, *AabHLH112*-RNAi transgenic plants were also generated and detected by genomic PCR. Specific detection primers were designed according to the sequences of *AabHLH112*, *OCS* terminator and *NptII* (kanamycin) genes. As shown in Supplementary Figures 5D,E, the partial *AabHLH112* sequence and *NptII* gene were detected in the *AabHLH112*-RNAi transgenic plants.

### AabHLH112 positively regulates the expression of *AaCPS*, *AaECS*, and *AaBFS* in *Artemisia annua*

**FIGURE 6:**
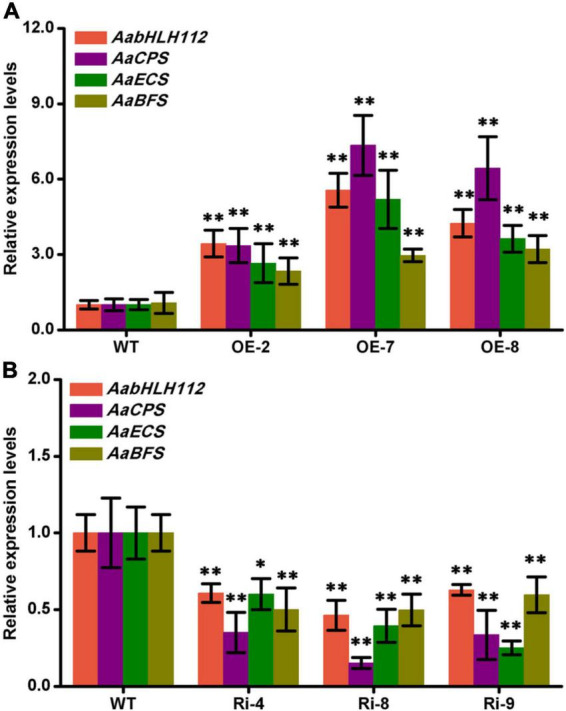
Genes relative expression levels in wild-type, *AabHLH112*-overexpression and *AabHLH112*-RNAi *Artemisia annua* plants. **(A)**: Relative expression levels of *AabHLH112*, *AaCPS*, *AaECS*, and *AaBFS* in wild type and *AabHLH112*-overexpression transgenic plants; **(B)**: Relative expression levels of *AabHLH112*, *AaCPS*, *AaECS*, and *AaBFS* in wild type and *AabHLH112*-RNAi transgenic plants. Error bars represent the standard deviations of three technical replicates. Statistical significance was assessed with Student’s *t*-test (*p < 0.05 and ^**^p < 0.01).

The expression level of *AabHLH112* in transgenic *A. annua* lines was determined by qPCR. Compared with the wild type (WT) *A. annua*, *AabHLH112* presented higher expression level in *AabHLH112*-overexpression lines of OE-2, OE-7, and OE-8 with 3.44-fold, 5.56-fold, and 4.24-fold higher, respectively (Figure 6A). Meanwhile, the expression levels of *AaCPS*, *AaECS*, and *AaBFS* in the three lines (OE-2, OE-7, and OE-8) were approximately 3.35-7.34 fold, 2.65-9.15 fold, and 2.24-2.55 fold higher compared to the WT respectively (Figure 6A). On the contrary, The expression level of *AabHLH112* were markedly decreased in *AabHLH112*-RNAi lines of Ri-4, Ri-8, and Ri-9 (Figure 6B), and the expression of *AaCPS*, *AaECS*, and *AaBFS* was also significantly reduced (Figure 6B) in the three *AabHLH112*-RNAi lines compared to WT plants. These results suggest that AabHLH112 positively regulates the expression of *AaCPS*, *AaECS* and *AaBFS* in *A. annua*.

### AabHLH112 positively regulates β-caryophyllene, *epi*-cedrol, and β-farnesene biosynthesis in *Artemisia annua*

**FIGURE 7:**
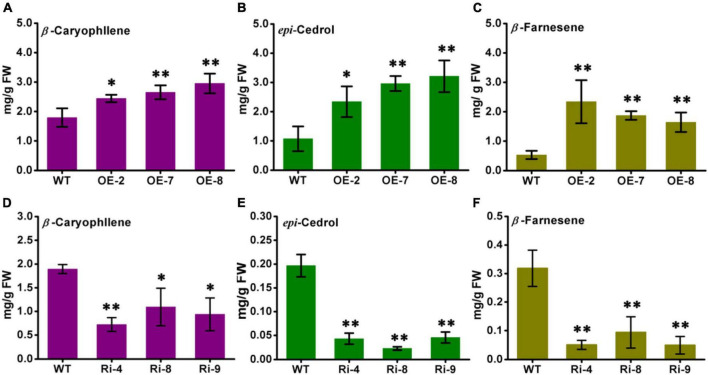
The contents of β-caryophyllene, *epi*-cedrol, and β-farnesene in wild-type, *AabHLH112*-overexpression, and *AabHLH112*-RNAi Artemisia annua plants. **(A)** β-caryophllene, **(B)** epi-cedrol, and **(C)** β-farnesene in *AabHLH112*-overexpressing lines, WT, wild type; OE-2,OE-7 and OE-8 are independent lines of *AabHLH112*-overexpressing lines; **(D)** β-caryophllene, **(E)** epi-cedrol and **(F)** β-farnesene in *AabHLH112*-RNAi lines, WT, wild type; Ri-4, Ri-8, and Ri-9 are independent lines of *AabHLH112*-RNAi lines, the data represents the means ± SD (*n* = 3), *p < 0.05, **p < 0.01 in student’s *t*-test.

The contents of β-caryophyllene, *epi*-cedrol, and β-farnesene in the *AabHLH112*-overexpression and RNAi transgenic lines were detected by the GC-MS method. The wild type *A. annua* produced 1.87 mg/g FW (fresh weight) of β-caryophyllene, 1.07 mg/g FW of *epi*-cedrol and 0.53 mg/g FW of β-farnesene, while the three independent *AabHLH112*-overexpression lines (OE-2, OE-7, and OE-8) exhibited significant increase in sesquiterpenes contents. OE-2, OE-7, and OE-8 contained β-caryophyllene at levels of 2.34 mg/g FW, 2.45 mg/g FW, and 2.95 mg/g FW, respectively, representing 25.13, 31.01, and 57.75% increases in content compared with WT plants (Figure 7A). Compared to WT plants, the content of *epi*-cedrol was increased by 118, 176, and 200% in the three *AabHLH112*-overexpression lines respectively (Figure 7B); and the content of β-farnesene was increased by 341, 253, and 201% in the three *AabHLH112*-overexpression lines (Figure 7C). In the *AabHLH112*-RNAi lines of Ri-4, Ri-8 and Ri-9, the contents of β-caryophyllene, *epi*-cedrol and β-farnesene were significantly reduced, compared to that in WT plants. Ri-4, Ri-8, and Ri-9 lines contained β-caryophyllene at levels of 0.73 mg/g FW, 1.09 mg/g FW, and 0.94 mg/g FW respectively, representing 61.91, 42.28, and 50.54% reduction in content compared with WT plants (Figure 7D). *Epi*-cedrol content was reduced by 78.12, 88.45, and 76.82% in Ri-4, Ri-8 and Ri-9 lines respectively, relative to that in WT plants (Figure 7E). β-farnesene content in Ri-4, Ri-8, and Ri-9 lines was reduced by 84.27, 70.55, and 84.58% respectively, compared with that in WT plants (Figure 7F). These results showed that AabHLH112 is a positive regulator of β-caryophyllene, *epi*-cedrol, and β-farnesene biosynthesis in *A. annua*.

## Discussion

*Artemisia annua* is a traditional Chinese medicinal plant, not only producing the well-known drug artemisinin, but also producing other important sesquiterpenes of medicinal value, such as β-caryophyllene and *epi*-cedrol. The bHLH TFs are the second largest transcription factor family in the plants, which play an important role in plant growth, development, and secondary metabolism. Previous research revealed that bHLH TFs play an important part in the regulation of terpenoids biosynthesis. In tomato, overexpression of CubHLH1 resulted in a significant increase in carotenoid content (Endo et al., 2016). MYC, one subgroup of bHLH family, plays an important role in JA, ABA, and GA signaling and is involved in the regulation of terpenoids biosynthesis in plants (Hong et al., 2012; Du et al., 2017). In *A. thaliana*, *AtMYC2* could directly bind to promoters of the sesquiterpene synthase genes *TPS21* and *TPS11* and activate their expression, thus increasing sesquiterpenes biosynthesis (Hong et al., 2012). *AaMYC2* overexpression could activate the transcription of *CYP71AV1* and *DBR2*, resulting in an increase of artemisinin content in *A. annua* (Shen et al., 2016). Besides, AabHLH1 positively regulated biosynthesis of artemisinin *via* activating the promoters of *ADS* and *CYP71AV1* (Ji et al., 2014). In this study, we found that AbHLH112 positively regulates the biosynthesis of β-caryophyllene, *epi*-cedrol, and β-farnesene in *A. annua* besides upregulating artemisinin biosynthesis.

The gene expression was synchronous with its product. In this study, we measured the expression levels of *AabHLHH112*, *AaCPS*, *AaECS*, and *AaBFS* in roots, stems, leaves and flowers. And we found that *AabHLH112* has the highest expression in leaves and flowers and the lowest expression in stems and roots, which has a similar expression pattern with *AaADS* involved in artemisinin biosynthesis (Pu et al., 2013). *AaCPS* and *AaBFS*, involved in β-caryophyllene and β-farnesene biosynthesis respectively, have a tissue-specific expression pattern, with the highest expression in flowers and the lowest expression in roots, stems and leaves. By contrast, *AaECS* is highly expressed in leaves and lowly expressed in roots and flowers, showing a tissue difference in the expression of these genes.

Methyl jasmonate (MeJA) is one of the most potent elicitors that can induce overaccumulation of many natural products in plants, such as artemisinin, tanshinones, vinblastine, and so on (Xiang et al., 2015; Shi et al., 2016; Zhang et al., 2018). Later studies showed that exogenous MeJA activated the transcription of some genes leading to the products’ overaccumulation (Wang et al., 2020). In this study, the 1-month-old *A. annua* seedlings were treated with MeJA for up to 24 h and the four genes expression were quantified by qPCR. The results showed that the genes detected had a higher expression after the MeJA treatment, indicating that exogenous MeJA treatment can induce the expression of sesquiterpene biosynthesis genes in *A. annua*. In addition, we found that the expression level of *AabHLH112* was induced as early as 3 h after the treatment, and then began to decline, while the highest expression levels of *AaECS* and *AaCPS* were detected at 12 h after MeJA treatment. This result suggested that these sesquiterpenes synthase genes might be regulated by *AabHLH112*. To further explore the function of AabHLH112 in the regulation of sesquiterpene biosynthesis genes expression, dual-LUC assay was taken to determine the activation of *AaCPS*, *AaECS*, and *AaBFS* promoters by *AabHLH112*. The results indicated AabHLH112 can significantly enhance the transcriptional activity of *AaCPS*, *AaECS*, and *AaBFS* promoters in *N. benthamiana* leaves (Figures 4B–D).

It is known that G-box is a putative recognition site for bHLH TFs. We have reported in a previous study that AabHLH112 can bind to the G-box in the *AaERF1* promoter and enhanced its transcription to upregulate artemisinin biosynthesis (Xiang et al., 2019). In this study, yeast one-hybrid assay (Y1H) showed that AabHLH112 can directly bind to pAaCPS-G3 and pAaECS-G1 fragments containing the G-box in the promoters of *AaCPS* and *AaECS*, but not bind to pAaCPS1-G1, pAaCPS-G2, pAaCPS-G4, pAaECS-G2, pAaECS-G3, and pAaBFS-G1, pAaBFS-G2, pAaBFS-G3 fragments (Figures 5B,D,F). Based on these results, we estimated that the flanking sequence of G-box *cis-*elements may affect the recognition of AabHLHL112 protein. In addition, there are three G-box *cis*-elements in the pAaCPS-G3 fragment and they are very close to each other. So, a profound study should be carried out to analyze which G-box is the real recognition site by AabHLH112. Interestingly, AabHLH112 positively regulated *AaBFS* expression to increase β-farnesene accumulation, but Y1H result showed there was no direct interaction between AabHLH112 and *AaBFS* promoter, suggesting that AabHLH112 upregulated β-farnesene biosynthesis through an indirect pathway, which deserves further exploration.

**FIGURE 8:**
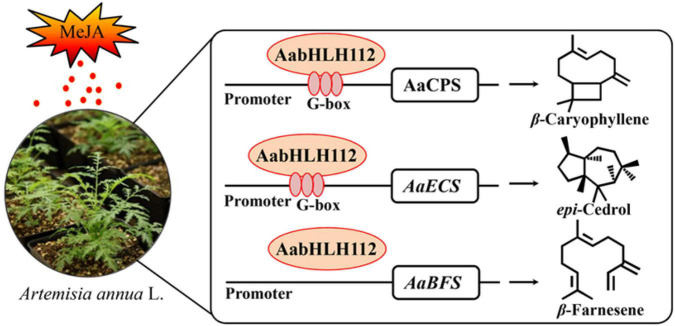
A simplified model for the regulation of sesquiterpenes biosynthesis by AabHLH112 in *Artemisia annua*.

Taken together, our study indicated that AabHLH112, which is induced by MeJA, positively regulates the expression of *AaCPS*, *AaECS*, and *AaBFS* through directly binding to their promoters or an indirect pathway, thereby enhancing the biosynthesis of β-caryophyllene, *epi*-cedrol, and β-farnesene in *Artemisia annua* (Figure 8). Our study revealed the mechanism of AabHLH112-mediated upregulation of sesquiterpenes biosynthesis, and provided a potentially useful transcription factor that could be used to improve sesquiterpenes production in *A. annua*.

## Data availability statement

The original contributions presented in this study are included in the article/Supplementary material, further inquiries can be directed to the corresponding author/s.

## Author contributions

LX, ZL, and YT conceived and coordinated the study and wrote the manuscript. LX performed the gene cloning, vector construction, and plant transformation work. GS, MY, and MW did the gene expression, luciferase analysis, and Y1H experiments. GS, XL, and PH helped with the metabolites analysis by GC-MS. All authors reviewed the results and approved the final version of the manuscript.

## Footnotes

1
http://bioinformatics.psb.ugent.be/webtools/plantcare/html/
