# Supplementary material for: AabHLH112, a bHLH transcription factor, positively regulates sesquiterpenes biosynthesis in Artemisia annua
Source: Front Plant Sci. 2022 Sep 2;13:973591. doi: 10.3389/fpls.2022.973591 (PMC9478121; doi:10.3389/fpls.2022.973591)
Supplement: Supplementary file 1 [file Data_Sheet_1.docx]

Supplementary Information

Article title: AabHLH112, a bHLH transcription factor, positively regulates sesquiterpene of *β*-caryophyllene, *epi*-cedrol and *β-*farnesene biosynthesis in *Artemisia annua*


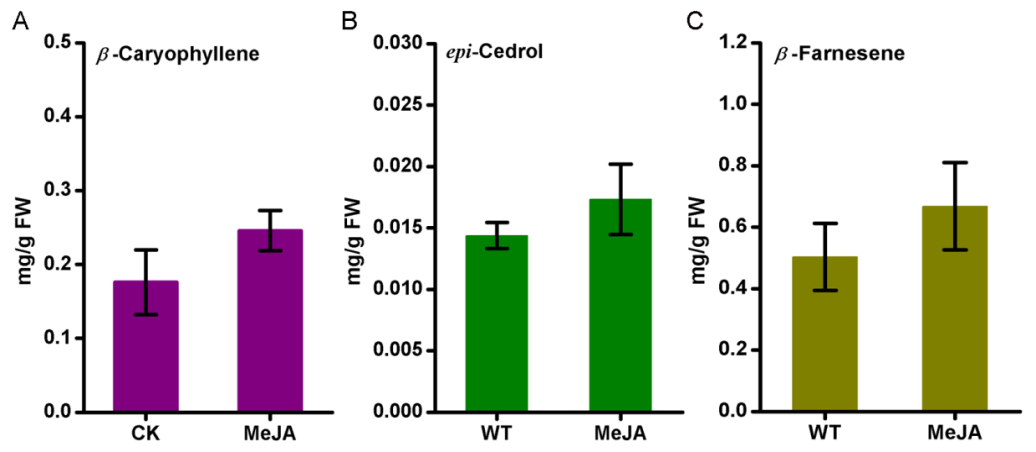


Fig.S1. The contents of *β*-caryophyllene, *epi*-cedrol and *β*-farnesene affected by MeJA treatment after 24h.


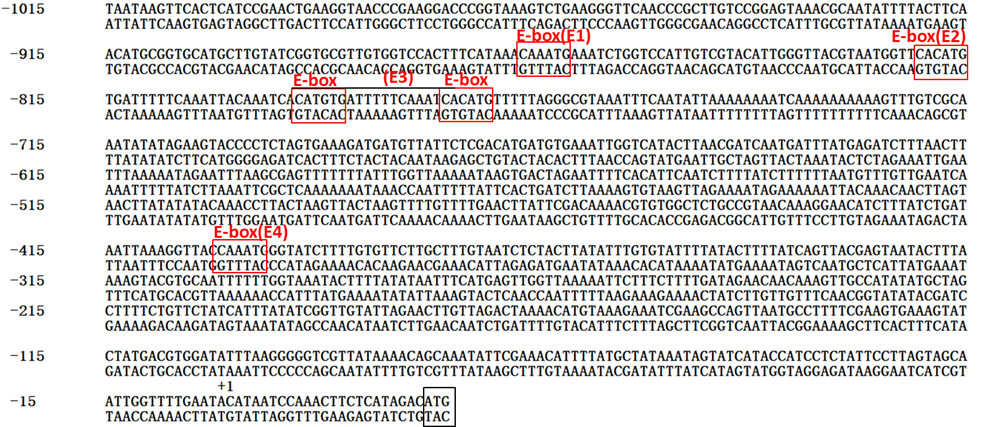


Fig.S2 The promoter sequence of *AaCPS.* The red boxes marked the predicted G-box *cis*-elemen.


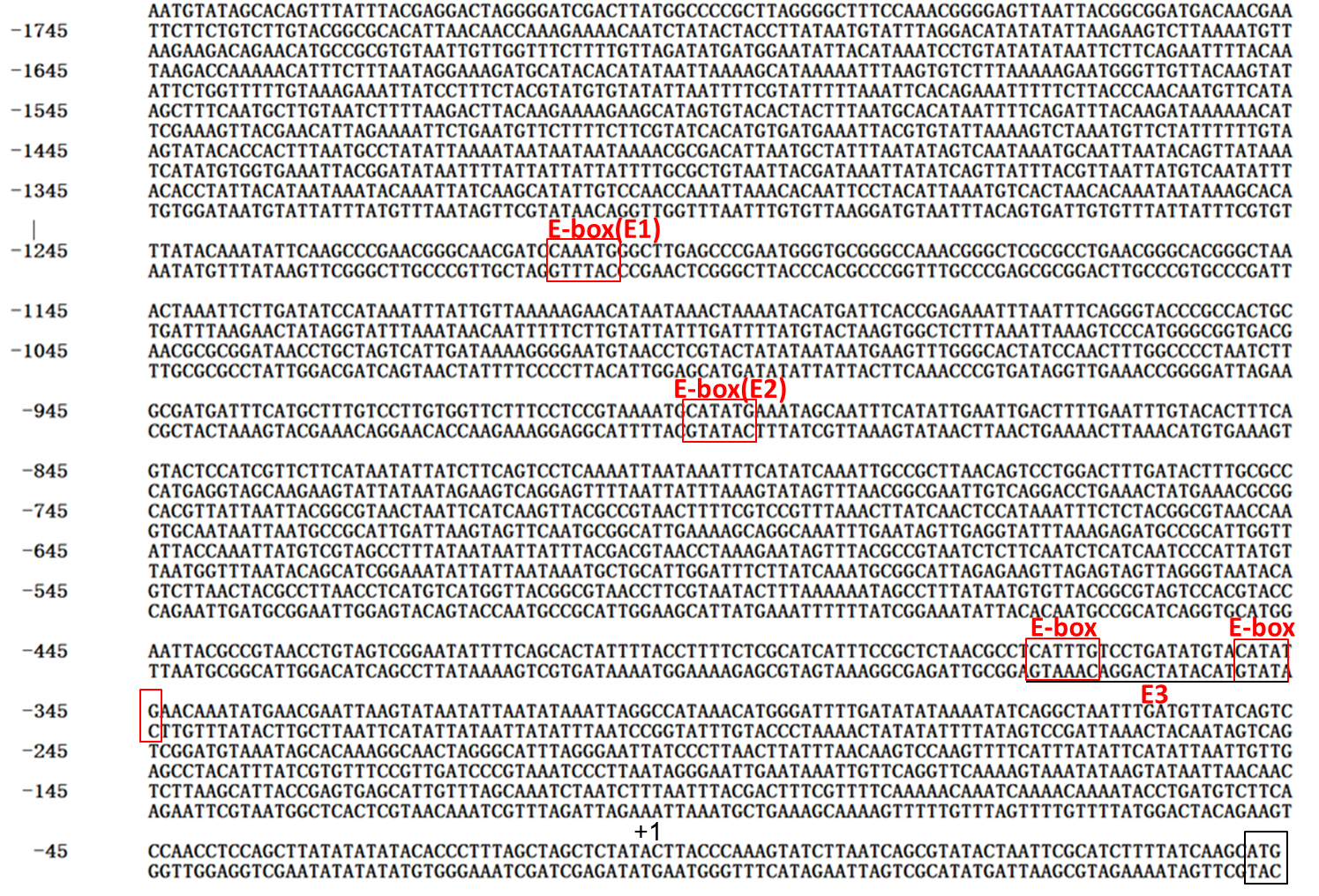


Fig. S3 The promoter sequence of *AaECS*. The red boxes marked the predicted G-box *cis*-element


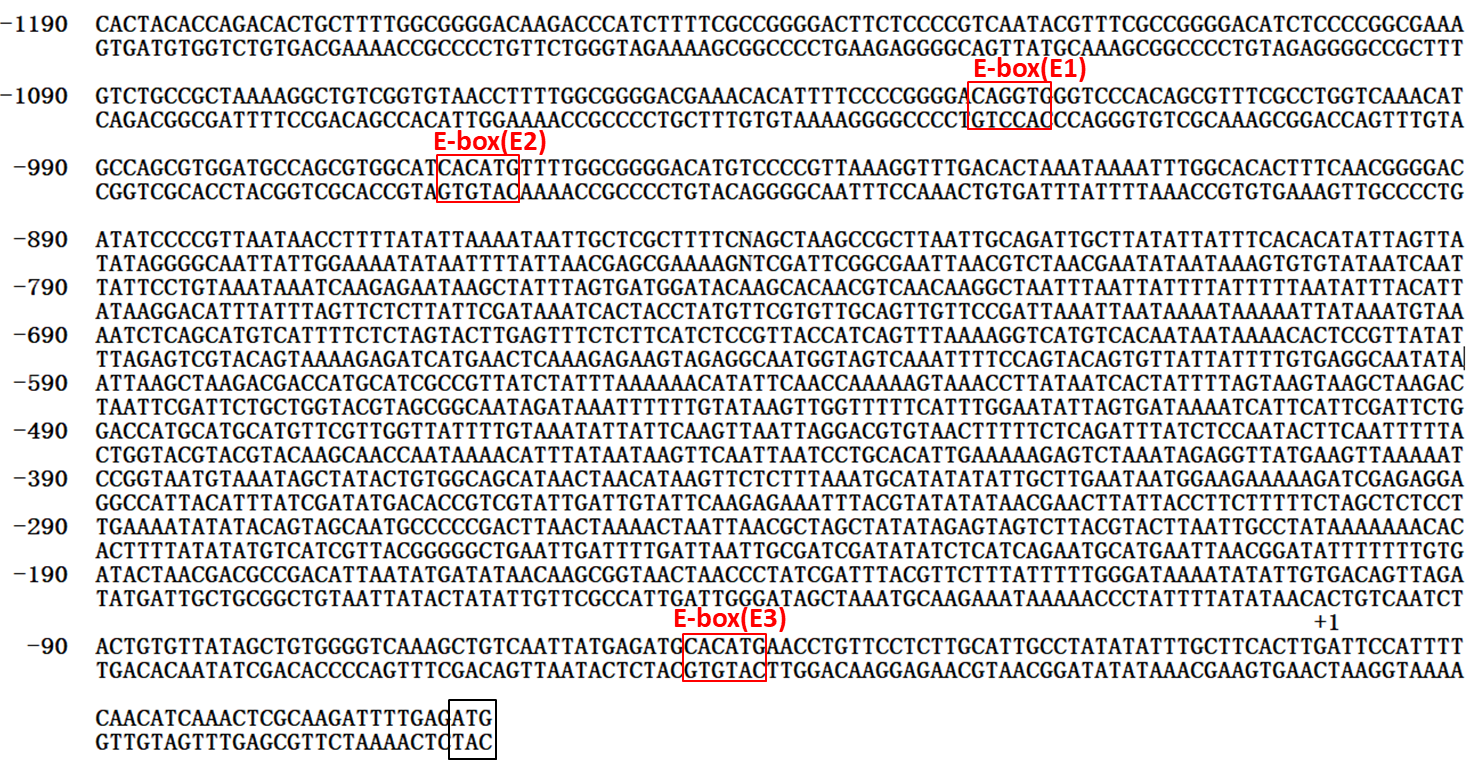


Fig. S4 The promoter sequence of AaBFS. The red boxes marked the predicted G-box cis-element


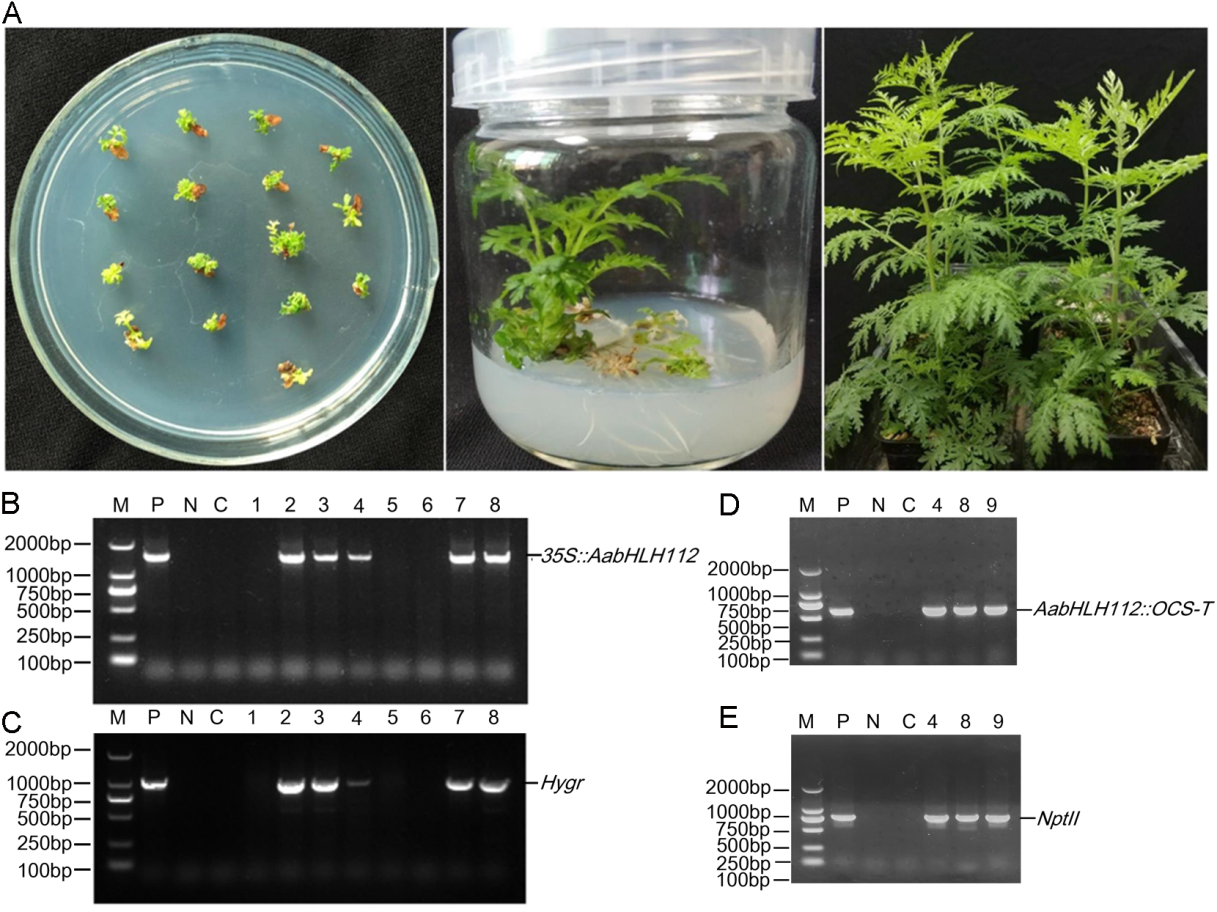


Fig. S5 Regeneration and molecular detection of AabHLH112-overexpression and AabHLH112-RNAi transgenic *A. annua*. (A): Regeneration of transgenic plants; (B): PCR detection of 35S::AabHLH112 gene; (C): PCR detection of *hygr* (Hygromycin B) gene in *AabHLH112*-overexpression plants; (D): PCR detection of *AabHLH112*::*OCS-T* gene in RNAi lines; (E): PCR detection of *NptII(*Kanamycin) gene in RNAi lines; M: DL2000 DNA marker; P: positive control; N: negative control; C: wild type *A. annua*.

Table S1 DNA sequence of oligonucleotide primers

| Primer | Sequence(5'-3') | Purpose |
| --- | --- | --- |
| AabHLH112F | TATGCTTAGGTCAGTGGT | RT-Q-PCR |
| AabHLH112R | GCTTCAAGTTCCTGGTTA | RT-Q-PCR |
| Ri112-1F | TCAACAGCATCTTCATGTTCAC | RNAi |
| Ri112-1R | GC TCTAGAACCAGTAGGAGTAAAACCCTC | RANi |
| Ri112-2F | TTTCCTTACCAATTGG TCTAGATCAACAGCATCTTCATGTTCAC | RANi |
| Ri112-2R | CATTTGGAGAGGACACGCTCGAGACCAGTAGGAGTAAAACCCTC | RANi |
| qAaECSF | CCTACGAATCACTCAACA | RT-Q-PCR |
| qAaECSR | TATCACCTCTACTCCAACA | RT-Q-PCR |
| qAaCPSF | AACAACAAGCATCTCATA | RT-Q-PCR |
| qAaCPSR | ACATTCAACCATTCTATCT | RT-Q-PCR |
| qAaBFSF | TTCATTCTCTTCATCTACG | RT-Q-PCR |
| qAaBFSR | ATCTTCTGGCTCATCATA | RT-Q-PCR |
| qAaActinF | CCAGGCTGTTCAGTCTCTGTAT | RT-Q-PCR |
| qAaActinR | AACTGCAGCGCTCGGTAAGGATCTTCATCA | RT-Q-PCR |
| proECSF | CGGGATCCACGAAAGATAATAAAGCATCGAC | Dual-LUC |
| proECSR | TATAGAGCTAGCTAAAGGGTGT | Dual-LUC |
| proCPSF | GGGGCCATTTGTACTAACCAGCCTAGC | Dual-LUC |
| proCPSR | AACTGCAGTATTCAAAACCAATTGCTACTAAGG | Dual-LUC |
| proBFSF | CACTACACCAGACACTGCTTTTGG | Dual-LUC |
| proBFSR | ATGGAATCAAGTGAAGCAAATATATAG | Dual-LUC |
| OE112F | CGGGATCCATGTTGTCTAGAGTTAATAGTG | overexpression |
| OE112R | CGAGCTCTCAAGCGACACCATGGTAACCTG | overexpression |
| F35S | ACTATCCTTCGCAAGACCCT | Detection |
| HygrF | AGTACTTCTACACAGCCATCGG | Detection |
| HygrR | TCCGGAAGTGCTTGACATTGG | Detection |
| JCbHLH112F | TCAACAGCATCTTCATGTTCAC | Detection |
| OCS-T | TGGCGCTCTATCATAGATGTC | Detection |
| NptIIF | ATGATTGAACAAGATGGATTG | Detection |
| NptIIR | TCAGAAGAACTCGTCAAGAAG | Detection |
| pCPSG1F | GTGGTCCACTTTCATAAACAAATGAAATCTGGTCCATTGTCGTAC | Yeast one-hybrid |
| pCPSG1R | GTACGACAATGGACCAGATTTCATTTGTTTATGAAAGTGGACCAC | Yeast one-hybrid |
| pCPSG2F | TTGGGTTACGTAATGGTTCACATGTGATTTTTCAAATTACAAATC | Yeast one-hybrid |
| pCPSG2R | GATTTGTAATTTGAAAAATCACATGTGAACCATTACGTAACCCAA | Yeast one-hybrid |
| pCPSG3F | CAAATTACAAATCACATGTGATTTTTCAAATCACATGTTTTTAGG | Yeast one-hybrid |
| pCPSG3R | CCTAAAAACATGTGATTTGAAAAATCACATGTGATTTGTAATTTG | Yeast one-hybrid |
| pCPSG4F | AACATCTTTATCTGATAATTAAAGGTTACCAAATGGGTATCTTTT | Yeast one-hybrid |
| pCPSG4R | AAAAGATACCCATTTGGTAACCTTTAATTATCAGATAAAGATGTT | Yeast one-hybrid |
| pECSG1F | AAGCCCGAACGGGCAACGATCCAAATGGGCTTGAGCCCGAATGGG | Yeast one-hybrid |
| pECSG1R | CCCATTCGGGCTCAAGCCCATTTGGATCGTTGCCCGTTCGGGCTT | Yeast one-hybrid |
| pECSG2F | TGGTTCTTTCCTCCGTAAAATGCATATGAAATAGCAATTTCATAT | Yeast one-hybrid |
| pECSG2R | ATATGAAATTGCTATTTCATATGCATTTTACGGAGGAAAGAACCA | Yeast one-hybrid |
| pECSG3F | TCTAACGCCTCATTTGTCCTGATATGTACATATGAACAAATATGA | Yeast one-hybrid |
| pECSG3R | TCATATTTGTTCATATGTACATATCAGGACAAATGAGGCGTTAGA | Yeast one-hybrid |
| pBFSG1F | AGCTGTCAATTATGAGATGCACATGAACCTGTTCCTCTTGCATTGCCTATAT | Yeast one-hybrid |
| pBFSG1R | ATATAGGCAATGCAAGAGGAACAGGTTCATGTGCATCTCATAATTGACAGCT | Yeast one-hybrid |
| pBFSG2F | CGTGGATGCCAGCGTGGCATCACATGTTTTGGCGGGGACATGTCCCCGTTA | Yeast one-hybrid |
| pBFSG2R | TAACGGGGACATGTCCCCGCCAAAACATGTGATGCCACGCTGGCATCCACG | Yeast one-hybrid |
| pBFSG3F | AACACATTTTCCCCGGGGACAGGTGGGTCCCACAGCGTTTCGCCTGGTCA | Yeast one-hybrid |
| pBFSG3R | TGACCAGGCGAAACGCTGTGGGACCCACCTGTCCCCGGGGAAAATGTGTT | Yeast one-hybrid |
| proCPSF1 | ACTTTATCCCTCTAGAGCTCC | promoter clone |
| proCPSF2 | ATAATAAGTTCACTCATCCGAAC | promoter clone |
| proCPSR1 | AGACGTTGGATAGCATCAATAAG | promoter clone |
| proCPSR2 | ACTCGTTGACTACTTGTTCTAC | promoter clone |
| proECSF1 | AATGTATTTAGGACATATATATTAAGAAG | promoter clone |
| proECSF2 | ACATTTCTTTAATAGGAAAGATG | promoter clone |
| proECSR1 | TAGTTCGCTCTTCACTTCTTCTTTC | promoter clone |
| proECSR2 | TCTTGCTCATCCTGGTCATAGG | promoter clone |
| proBFSF1 | ACACATATAATTAGTTATATTCC | promoter clone |
| proBFSF2 | CACTACACCAGACACTGCTTTTGG | promoter clone |
| proBFSR1 | TTGAAATATGATATACAATTCCAAC | promoter clone |
| proBFSR2 | AATTGATCTCCCCATATGCTAG | promoter clone |
|  |  |  |
